# Barriers and Willingness to Accept Re-Employment among Unemployed Senior Workers: The SeniorWorkingLife Study

**DOI:** 10.3390/ijerph17155358

**Published:** 2020-07-25

**Authors:** Kristina Thomassen, Emil Sundstrup, Sebastian V. Skovlund, Lars L. Andersen

**Affiliations:** National Research Centre for the Working Environment, DK-2100 Copenhagen, Denmark; ESU@nfa.dk (E.S.); svs@nfa.dk (S.V.S.); LLA@nfa.dk (L.L.A.)

**Keywords:** seniors, unemployment, return to work, occupational groups, labor market

## Abstract

Labor market participation has a positive impact on social inclusion and is linked to financial security. This study identifies barriers and willingness to accept re-employment among unemployed seniors that could highlight opportunities for societal action. From the first wave of SeniorWorkingLife in 2018 combined with the Danish version of the International Standard Classification of Occupations register (ISCO), +50-year-old unemployed senior workers (*n* = 1682) were stratified into mainly seated work (ISCO 1–4) and mainly physical work (ISCO 5–9), respectively, in their latest employment. We used SurveyFreq and SurveyLogistics of SAS combined with model-assisted weights based on national registers to estimate representative frequencies and odds ratios (OR) for barriers and willingness to accept re-employment. Higher age was perceived as a general barrier for re-employment in both groups. Health was a more pronounced barrier for seniors with mainly physical work compared to seniors with mainly seated work (OR 2.35; CI95 1.31–4.21). Overall, seniors showed a large degree of flexibility and willingness to re-enter the labor market. Different barriers and willingness to accept re-employment exist among currently unemployed seniors. These results highlight the need for different approaches across occupational groups to help unemployed seniors back into the labor market.

## 1. Introduction

Being a part of the labor market—even at a older age—is important for financial security, and it has a positive impact on social inclusion and self-rated health [1,2]. However, re-employment among currently unemployed seniors is not always without challenges.

Once unemployed, seniors (+50 years) experience more difficulty re-entering the labor market compared with younger job seekers and are, therefore, at increased risk of becoming long-term unemployed [3,4]. Long-term un-employment also decreases the chances of re-entering the labor market [5,6]. Previous literature shows that employers are more likely to hire workers who have not been long-term unemployed as long term unemployment illustrates possible unwanted characteristics or lack of motivation [6]. This can induce a vicious circle that is hard to break. Seniors’ difficulties re-entering the labor market are often characterized by negative assumptions. Age discrimination and comprehension about seniors’ ability to work have been mentioned as an important barrier to re-employment [1,7,8], although seniors may be more productive, loyal and experienced [3,8]. The likelihood of returning to the labor market also depends on the seniors’ health since poor health before unemployment decreases the likelihood of returning to the labor market [9]. Other pronounced barriers to re-employment include lack of training opportunities and support for job search [4,10,11]. While the likelihood of returning to the labor market decreases with long-term unemployment, an early and coordinated effort—e.g., support by job centers (Job center: “The jobcenter is charged with procuring jobs for jobseekers and ensuring that companies find the labor they need. The jobcenter assists all applicants in finding help and guidance on recruitment, job hunting or general information on the labor market” [12])—has a positive effect on re-employment [13,14,15].

The changing demographics towards a growing proportion of elderly people makes the older population an increasingly important part of the total workforce, with an important economic contribution to society [1]. Therefore, several countries are developing policies to encourage elderly people to stay longer in the labor market [16]. The high level of unemployment among senior workers in many countries shows that further action is needed [17]. In this regard, exploring the possibilities of re-employment and elucidating what barriers and possibilities exist among unemployed seniors to re-enter the labor market are vital. Gender differences are also important to consider as women in general retire earlier than men. Men and women are often employed in different occupations, which may affect the possibilities for re-employment [18].

Like the general population, unemployed seniors are a heterogeneous group, and factors such as previous work characteristics, gender and age may influence both barriers and willingness to accept re-employment [19]. Thus, this study aimed to determine factors influencing re-employment among unemployed seniors across different occupational groups in Denmark. We hypothesize that there is an association between previous work characteristics of unemployed seniors and factors influencing re-employment. Shedding light on perceptions of barriers and willingness to accept re-employment among different groups of unemployed seniors can help guide societal action plans.

This study draws on data from the first wave (2018) of the SeniorWorkingLife study, which include a representative sample of +50-year-old currently unemployed people in Denmark.

## 2. Material and Methods

### 2.1. Material and Methods

#### 2.1.1. Study Design and Study Population

The present study is based on the first wave of SeniorWorkingLife where questionnaires were sent to 30,000 participants +50-years between July and October 2018 [20]. This included both employed and unemployed, as well as people receiving voluntary early retirement benefits and disability benefits. The present article draws on data from 1576 unemployed individuals. The purpose of SeniorWorkingLife is to identify a wide range of push and stay mechanisms for labor market participation [20]. Participants were randomly drawn by Statistics Denmark and invited to participate with a personal questionnaire-link via e-Boks (online digital mailbox linked to the Danish social security number). The survey data were merged with high-quality national registers through the unique social security number assigned to all Danish residents at birth or immigration. This has been described in more detail in the published protocol paper [20]. The study is registered as a cohort study in ClinicalTrials.gov (identifier: NCT03634410). A longitudinal follow-up will be ready by the end of 2020.

Not all participants answered all survey questions; thus, the exact number of participants varies.

#### 2.1.2. Subgroups

We extracted information on the participants’ latest employment to classify them into the International Standard Classification of Occupations (ISCO). All participants were stratified into nine occupational groups based on the official Danish version of the ISCO. Each ISCO group is based on specific requirements ranging from 1 (most basic) to 4 (most advanced). ISCO groups 1–4 are characterized by high skill levels and/or predominantly seated work (ISCO 1–4). ISCO groups 5–9 are characterized by physically demanding work, e.g., walking, standing, lifting, using arms, back and legs. The dichotomization was based on a previous analysis showing that the majority in ISCO groups 1–4 have seated work and the majority in ISCO groups 5–9 have physical work [16]. In the present population of unemployed seniors, a similar pattern was observed (Table 1). ISCO is described in detail elsewhere [16,20].

#### 2.1.3. Questionnaire about Barriers and Willingness to Accept Re-Employment

Participants were asked to answer two multiple-choice questionnaire batteries concerning (1) perceived barriers for re-employment and (2) willingness to accept re-employment. The first battery contained nine multiple-choice response options concerning barriers to re-employment: Choose the conditions that prevent or make it difficult for you to get a job: (1) your health, (2) your age, (3) your qualifications are somewhat outdated, (4) you are overqualified for the vacancies, (5) you have already set your mind to retire from the labor market, (6) you have family obligations, (7) you are trying to start your own business, (8) you are lacking a network of acquaintances who can help you to find vacancies, (9) none of the above.

The latter battery contained ten multiple-choice response options concerning willingness to accept re-employment: Which of the following conditions would you be willing to accept to get a job (compared to your most recent job)? (1) lower salary, (2) work-time reduction, (3) poorer working conditions, (4) take another job function, (5) doing work with less responsibility, (6) handle job tasks that are significantly below your competencies, (7) take a completely different job than your education, (8) move, (9) increased transport time compared to your previous job, (10) none of the above.

Participants could choose multiple answers in each battery without prioritization. The multiple-choice response options in both batteries were given in a random order for each participant, except for the last option “none of the above”, which was always the last option.

### 2.2. Statistics

The SurveyLogistics procedure (SAS, version 9.4 (SAS Institute Inc., Cary, NC, USA)) was used to model odds ratios (OR) and 95% confidence intervals for barriers and willingness to accept re-employment for senior workers with mainly seated and physically demanding work, respectively. For ISCO, groups 1–4 were used as reference, i.e., ORs for groups 5–9. For sex, men were used as reference, i.e., ORs for women. For age, the estimates indicate the OR’s of each one-year increase in age (continuous variable). These factors were entered in the statistical model simultaneously, i.e., they were mutually adjusted. To produce estimates of prevalence and 95% confidence intervals SurveyFreq procedures were used.

Due to the different size and response percentages of subgroups, model-assisted weights were used. These weights were used for both the SurveyFreq and SurveyLogistic procedures and were based on information from high-quality national registers at Statistics Denmark and took into account sex, age, occupational industry, highest completed education, family income, family type and origin. This ensured that the reported estimates are representative of unemployed +50-year-old people in Denmark.

## 3. Results

Table 1 illustrates demographics, work characteristics and ISCO group of the study sample of 1576 unemployed seniors. Mean age for ISCO groups 1–4 was 57.2 for woman and 57.1 for men. For ISCO groups 5–9 the mean age was 56.6 for woman and 57.8 for men.

Table 2 shows prevalence’s and odds ratios of perceived barriers to re-employment. Over half of the seniors perceived age as a barrier for re-employment (54%). Other general barriers were “Your qualifications are somewhat outdated” (26%), “Your health” (19%) and “You are lacking a network of acquaintances who can help to find to find vacancies” (17%). Mean age was approximately the same in both ISCO groups as well as for women and men. Our results show an age effect, and increasing age was perceived as a significant barrier to re-employment (OR 1.10; CI 1.03–1.19). When comparing groups, health was a significantly more pronounced barrier for seniors in ISCO 5–9 compared to seniors in ISCO 1–4 (OR 2.35; CI95 1.31–4.21). There was a significant association between seniors with previously seated jobs and perceiving to be overqualified as a barrier to re-employment (OR 0.47; CI95 0.23–0.98). We observed other significant differences, but the prevalence’s were low.

Table 3 shows prevalence’s and odds ratios of willingness to accept re-employment. Seniors in both groups were generally willing to accept several conditions. Around half of the seniors were willing to take another job function (53%) and to take a completely different job from their education (47%). Seniors were also willing to do work with less responsibility (43%) and accept work-time reduction (41%)

Physical characteristics of seniors’ previous job demands were associated with their willingness to work with less responsibilities (OR 0.35; CI95 0.22–0.55) and to handle job tasks significantly below their competencies (OR 0.51; CI95 0.31–0.84). Furthermore, did our results showed an association between previous job demands and seniors’ willingness to accept worktime reduction (OR 0.55; CI95 0.33–0.92) and lower salary (OR 0.28; CI95 0.16–0.50). Significant gender differences were in relation to work-time reduction, where women were more likely to accept work-time reduction compared to men (OR 1.63; CI95 1.01–2.63). We observed other significant differences, but the prevalence’s were low.

## 4. Discussion

In the analysis of 1576 unemployed seniors, we found several important possibilities for re-employment. Seniors’ willingness and barriers to return to work differed between those with a previous seated job and those with a previous physically demanding job. Although some general findings were obtained—e.g., age as a perceived barrier—the results also demonstrate that unemployed seniors are a heterogeneous group indicating the need for a differentiated approach benefitting seniors across occupational groups to re-enter the labor market.

Increasing age was in both groups perceived as a barrier to re-employment. Relating this result to retirement reasons, research shows a demographic characteristic such as age is a reason to retire as aging may in many ways affect the possibility to work [21]. Age could consequently be a reason to not wanting to get back into the labor market, fx due to lack of work motivation [8,22]. Older age is also stated to be linked to age discrimination and prejudice [8]. Research from Denmark reported that among unemployed workers +50 years, almost 25% had experienced age discrimination [8]. In the present study, we asked more broadly about age as a barrier for re-employment, and the underlying factors may be related to both seniors’ own assessment of their age and re-employment as well as to age discrimination. Employers’ negative attitudes towards senior workers are based on beliefs that seniors are slow and that learning new things is difficult [21]. These negative generalized beliefs do however not stand alone as it is argued that seniors are more reliable and experienced [21]. Generalized attitudes and beliefs towards seniors could have serious consequences for their employment opportunities, giving seniors a disadvantage to re-enter the labor market.

This study investigated factors influencing re-employment across different occupational groups.

Health was a significantly more pronounced barrier for seniors in ISCO groups characterized by physically demanding work. One possible explanation for these health differences may be the general characteristics of the populations. Health conditions vary among occupational groups, and workers with short education experience to a larger extent musculoskeletal disorders and sickness absence [23]. ISCO groups 1–4 are characterized by having on average longer education, and it was previously reported that health inequalities are related to sociodemographic determinants such as education and job function, i.e., less educated people have fewer years of good health [9]. Health issues in this study are unknown. It was however previously reported that persons, especially seniors, with poor health before unemployment, were less likely to return to the labor market [9,24]. These health differences could also be linked to seniors’ previous job demands. ISCO groups 5–9 are generally characterized by physically strenuous work and high physical work demands, and it has been reported that these workers would withdraw later from the labor market if the work was less physically strenuous [16]. Thus, the combination of poor health and hard physical work can be a major barrier—not only for those working—but also for those trying to get back into the labor market. Continued education and the role of the job centers may be a way forward to channel this particular group of senior workers into jobs that are less physically demanding. This may require re-training or development of certain skills to be able to handle other types of work tasks than usual. As discussed in the following, the willingness to “change track” is high among seniors.

The high prevalence’s illustrated in Table 3 indicate a widespread degree of flexibility and willingness among unemployed seniors across ISCO groups to compromise to return to the labor market. Most pronounced were seniors’ willingness to take another job (53%) and to take a completely different job from their education (47%). This should be supported by re-training and job-seeking support taking the full advantage of this workforce, especially when lacking a network of acquaintances was also reported as a barrier to re-employment. This is consistent with a previous study reporting that lacking a network with job contacts increases the duration of unemployment [6].

Furthermore, seniors in ISCO groups 1–4 reported being overqualified for the vacancies as a barrier to re-employment. They were however willing to handle job tasks that are significantly below their competencies and to do work with less responsibilities. These results indicate that many seniors are willing to compromise to return to the labor market. In stark contrast to seniors’ flexibility and willingness to re-enter the labor market, a study from Sweden showed that employers were believed to generalize about senior workers as not being flexible and lacking competence [21]. The same study also stated that almost half of the employers believed seniors not to be adaptable to changes at the workplace [21]. Negative assumptions about seniors have also been associated with employers age, as older employers tend to have a more positive attitude towards seniors, not only due to age difference but because older employers have more experience working with seniors [21]. Having a negative attitude towards seniors for not being flexible and lacking competence based on stereotypes is a serious matter and is impairing seniors’ possibility to re-enter the labor market. Taking advantage of seniors’ willingness to work could in a larger perspective lead to a more flexible working market, where some workers could fill in the gaps in occupations where there is a lack of available workforce.

We found that seniors’ willingness to accept different conditions is strongly related to their previous job. Seniors with mainly seated work (ISCO 1–4) were more likely to accept a lower salary compared to seniors with mainly physically demanding work (ISCO 5–9). ISCO groups 1–4 are—besides differences in physical work demands—also generally characterized by higher skill requirements, i.e., on average longer education and higher salary [16]. Thus, giving seniors in ISCO groups 1–4 more room to accept these conditions. This is consistent with findings from a previous study showing that those with longer education were more willing to compromise their salary to find a job compared to those without a higher degree [6]. Additionally, seniors in ISCO groups 1–4 were willing to accept work-time reduction, which again could be related to income as fewer working hours usually result in a lower salary.

Willingness to accept different working conditions for re-entering the labor market was also dependent on gender. Women were more likely to accept fewer working hours than men. Factors concerning gender-specific retirement reasons show that women are more likely to retire due to family reasons such as family care and spending time with grandchildren [25,26]. Thus, these family reasons could also be expected among unemployed seniors and explain our findings.

### Strengths and Limitations

Non-response is always a study limitation. This was, however, accounted for in the statistical analysis that was performed using model-assisted weights based on high-quality national registers, to produce representative estimates of +50-year-old unemployed seniors in Denmark. Using the Danish version of ISCO, seniors were stratified into ISCO groups based on their latest occupation. This is an internationally accepted way to group occupations and eliminates any self-report bias. However, self-report bias could have influenced the present results, as the responses are dependent on the perception of the unemployed senior replying to the questionnaire.

## 5. Conclusions

In conclusion, older age was a general barrier to re-employment. Health was a more pronounced barrier for seniors with physically demanding work. Seniors with mainly seated work were more likely to accept lower salaries and work-time reduction. Gender differences existed in relation to work-time reduction, where women were more likely to accept work-time reduction compared to men. Thus, different barriers and willingness to accept re-employment exist among currently unemployed seniors across occupational groups. These results indicate that there is a need for different approaches across occupational groups to help unemployed seniors back into the labor market.

## Figures and Tables

**Table 1:** Characteristics of the population and work characteristics in ISCO groups 1–4 (mainly seated) and 5–9 (mainly physically demanding work).

	ISCO 1–4 (Seated Work)		ISCO 5–9 (Physical Work)	
	Men	Women	Men	Women
Variable Label	n = 408	n = 622	n = 340	n = 312
Age, mean (SD)	57.1 (12.6)	57.2 (12.3)	57.8 (10.7)	56.6 (10.7)
Physical activity work, percentage (95% CI)				
Seated	60 (49–70)	65 (57–74)	31 (17–44)	23 (10–36)
Standing or walking	24 (15–34)	24 (16–32)	17 (10–23)	17 (9–26)
Standing or walking with a lot of lifting or carrying	12 (6–18)	7 (4–11)	33 (22–43)	38 (26–50)
Heavy or fast work that is physically strenuous	4 (0–9)	3 (0–6)	20 (10–30)	22 (10–34)

**Table 2:** Barriers to re-employment among men and women in ISCO groups 1–4 and 5–9. The first five columns are percentages of the respondents indicating this barrier, and the last three columns are OR’s and 95% CI. Significant associations are marked with bold.

		ISCO 1–4		ISCO 5–9		ISCO 5–9 vs. 1–4	Woman vs. Men	Age
	
	All	Men	Women	Men	Women	OR (95%CI)	OR (95%CI)	OR (95%CI)
	n = 1576	n = 389	n = 586	n = 311	n = 290			
Your age	54%	59%	59%	44%	53%	0.63 (0.39–1.02)	1.21 (0.76–1.94)	**1.10 (1.03** **–1.19)**
Your qualifications are somewhat outdated	26%	22%	23%	21%	26%	1.05 (0.62–1.79)	1.15 (0.69–1.91)	0.95 (0.91–1.00)
Your health	19%	15%	12%	31%	23%	**2.35 (1.31–4.21)**	0.72 (0.40–1.29)	1.00 (0.93–1.07)
You are lacking a network of acquaintances who can help you to find vacancies	17%	14%	16%	11%	27%	1.32 (0.74–2.36)	1.65 (0.94–2.91)	**0.94 (0.89** **–0.99)**
You are overqualified for the vacancies	15%	23%	14%	10%	9%	**0.47 (0.23** **–0.98)**	0.61 (0.34–1.11)	0.97 (0.92–1.02)
You have already set your mind to retire from the labor market	8%	7%	10%	7%	3%	0.61 (0.32–1.16)	1.40 (0.64–3.07)	**1.23 (1.13** **–1.33)**
You have family obligations	3%	3%	1%	3%	0%	0.86 (0.30–2.46)	**0.23 (0.07** **–0.82)**	0.78 (0.58–1.04)
You are trying to start your own business	3%	5%	4%	1%	1%	**0.29 (0.12** **–0.70)**	0.78 (0.25–2.40)	**0.82 (0.72** **–0.93)**
None of the above	19%	17%	16%	27%	24%	1.74 (0.91–3.33)	0.86 (0.46–1.61)	0.93 (0.84–1.03)

Adjusted for sex, age and ISCO group. Estimates are weighted for sex, age, occupational industry, highest completed education, family income, family type and origin.

**Table 3:** Willingness to accept re-employment among men and women in ISCO groups 1–4 and 5–9. The first five columns are percentage of the respondents indicating this willingness, and the last three columns are OR’s and 95% CI. Significant associations are marked with bold.

		ISCO 1–4		ISCO 5–9		ISCO 5–9 vs. 1–4	Woman vs. Men	Age
	
	All	Men	Women	Men	Women	OR (95%CI)	OR (95%CI)	OR (95%CI)
	n = 1576	n = 389	n = 586	n = 311	n = 290			
Take another job function	53%	59%	53%	39%	56%	0.72 (0.45–1.16)	1.01 (0.63–1.61)	**0.90 (0.85–0.95)**
Take a completely different job than you your education	47%	41%	52%	43%	51%	1.02 (0.64–1.63)	1.45 (0.92–2.29)	0.96 (0.91–1.01)
Doing work with less responsibility	43%	50%	55%	22%	34%	**0.35 (0.22** **–** **0.55)**	1.37 (0.86–2.19)	**0.94 (0.89–0.99)**
Work time reduction	41%	38%	52%	28%	35%	**0.55 (0.33–0.92)**	**1.63 (1.01–2.63)**	0.95 (0.91–1.00)
Lower salary	35%	52%	40%	18%	21%	**0.28 (0.16–0.50)**	0.68 (0.41–1.14)	**0.91 (0.87–0.96)**
Handle job tasks that are significantly below your competencies	32%	38%	37%	23%	23%	**0.51 (0.31–0.84)**	0.95 (0.59–1.54)	0.98 (0.93–1.03)
Increased transport time compared with your previous job	28%	37%	28%	15%	31%	0.63 (0.36–1.09)	0.92 (0.55–1.54)	**0.90 (0.85–0.95)**
Accept poorer working conditions	11%	18%	8%	8%	12%	0.74 (0.31–1.79)	0.57 (0.26–1.24)	0.94 (0.88–1.01)
Move	5%	6%	3%	10%	6%	2.08 (0.76–5.70)	0.47 (0.18–1.19)	**0.89 (0.81–0.97)**
None of the above	19%	18%	15%	22%	22%	1.56 (0.82–2.97)	1.04 (0.55–1.98)	**1.22 (1.11** **–1.35)**

Adjusted for sex, age and ISCO group. Estimates are weighted for sex, age, occupational industry, highest completed education, family income, family type and origin.
